# Synthetic diversity in the preparation of metallic uranium

**DOI:** 10.1098/rsos.211870

**Published:** 2022-03-23

**Authors:** Harry Jang, James Louis-Jean, Bradley Childs, Kiel Holliday, Dallas Reilly, Matthew Athon, Kenneth Czerwinski, David Hatchett, Frederic Poineau

**Affiliations:** ^1^ Department of Chemistry and Biochemistry, University of Nevada, Las Vegas, NV, USA; ^2^ Materials Science Division, Lawrence Livermore National Laboratory, Livermore, CA, USA; ^3^ Pacific Northwest National Laboratory, Richland, WA, USA

**Keywords:** uranium, metal, preparation, reduction, electrochemistry, decomposition

## Abstract

Uranium metal is associated with several aspects of nuclear technology; it is used as fuel for research and power reactors, targets for medical isotope productions, explosive for nuclear weapons and precursors in synthetic chemistry. The study of uranium metal at the laboratory scale presents the opportunity to evaluate metallic nuclear fuels, develop new methods for metallic spent fuel reprocessing and advance the science relevant to nuclear forensics and medical isotope production. Since its first isolation in 1841, from the reaction of uranium chloride and potassium metal, uranium metal has been prepared by solid-state reactions and in solution by electrochemical, chemical and radiochemical methods. The present review summarizes the methods outlined above and describes the chemistry associated with each preparation.

## 1. Introduction

Uranium, element 92, is the highest atomic number radioelement naturally occurring in significant quantities. It was isolated in 1789 by M. H. Klaproth from pitchblende mineral, and its radioactivity was discovered by H. Becquerel in 1896 [1,2]. Prior to its isolation, uranium has been used to colour glass and ceramics [3]. The contemporary uses of uranium rely on the nuclear properties of ^235^U which is the only fissile naturally occurring uranium isotope. The isotopes ^238^U, ^235^U and ^234^U with respective isotopic abundances of approximately 99.274%, approximately 0.720% and approximately 0.005% represent the natural composition of uranium. Owing to the relatively low concentration of ^235^U in nature, the enrichment of uranium is required for most nuclear applications [4,5].

*In situ* leaching and underground/open-pit mining are two methods for uranium mining. In the former, a leaching solution is used to dissolve ore minerals, and uranium is extracted from the resulting solution. In the latter, the ore material is ground, chemically leached and thermally converted to U_3_O_8_. In 2019, two-thirds of the world's production of uranium originated from Kazakhstan, Canada and Australia [6].

Following the discovery of nuclear fission in the 1930s, uranium has been used as nuclear fuel for energy production and as explosives for nuclear devices. The first nuclear reactor was started in 1942, while the first nuclear weapon was used in 1945 [7]. In both technologies, uranium in the form of metal was used as the energy source. It is noted that CP-1 (Chicago Pile-1), the first reactor, used uranium oxide and uranium metal as a fuel.

Today, uranium metal is still associated with weapon applications and energy production, but it is also used as munitions for high penetrating bullets, fuel for research reactors, targets for medical isotope production and precursors in synthetic chemistry [8–11]. Interest in metallic fuel has risen owing to nuclear material proliferation concerns [12–15] and its applications in commercial light water reactors such as material for fuel rods [16,17]. It is noted that the uranium material is not systematically enriched for nuclear applications, for example, fuels for uranium-graphite-gas reactors consist of natural uranium, while uranium bullets consist of depleted uranium.

The study of uranium metal at the laboratory scale presents the opportunity to examine metallic nuclear fuels, develop new methods for metallic spent fuel reprocessing and advance the science relevant to nuclear forensics. For example, morphological features and impurities in uranium materials may reveal historical information and provide nuclear forensics signatures (i.e. origin of uranium, preparation, purification and enrichment methods) [18–20].

Uranium metal was first isolated in 1841 by E. Peligot from the reaction of chlorine gas with carbon and UO_2_, followed by the thermal treatment of the resulting UCl_4_ product with potassium metal [7,21,22]. Historically, the first preparation of uranium metal by electrochemical method was performed in 1893 from the electrolysis of molten Na_2_UCl_6_ using a carbon electrode under hydrogen atmosphere [23]. In the ninteenth century, little research was performed on the production of uranium metal. It was not until World War II that methods for the large-scale production of uranium metal were actively pursued.

Production of uranium metal is challenging owing to its pyrophoricity and the multistep refinements required. While some of the challenges have been resolved for large-scale productions, the preparation of uranium metal at the laboratory scale (0.1–1 g) is still challenging. High temperature chemical reductions of uranium halides and uranium oxides by group II metals are the predominant methods for uranium metal preparation and have been used for decades. The industrially common method used to produce large amounts of uranium metal is the reduction of UF_4_ with magnesium (magnesiothermic reduction (MTR)) or calcium metal (calciothermic reduction). Though not as common, reductions with carbon (carbothermic reduction) have also been used. Uranium metal was also prepared by metallothermic reductions with various metals (e.g. Li [24], Na [21,25,26], K [21,22]). Uranium metal can be prepared by electrochemical reductions at high temperatures in molten salts and at room temperature in room temperature ionic liquids (RTILs), acetic acid and organic solvents. Other preparation methods include thermal decompositions using arc melter and laser and radiochemical reductions using Co-60 sources.

While a chronology of uranium metal production methods up to the 1960s has been well detailed [21] and recent reviews about preparation using metallothermic reductions have been published [9,27], to our knowledge no review covering the entire synthetic chemistry of uranium metal has been reported. Here, we summarize the methods that have been used to prepare uranium metal from the beginning of the twentieth century through to the present day. The review outlines the diversity of the methods used, including solid-state reactions, electrochemical, chemical and radiochemical methods along with thermal decompositions. The physico-chemistry behind each preparation is presented and the reaction yield, nature of the product metal and byproduct impurities are reported.

## 2. Physico-chemical properties of uranium metal

**Figure 1.**
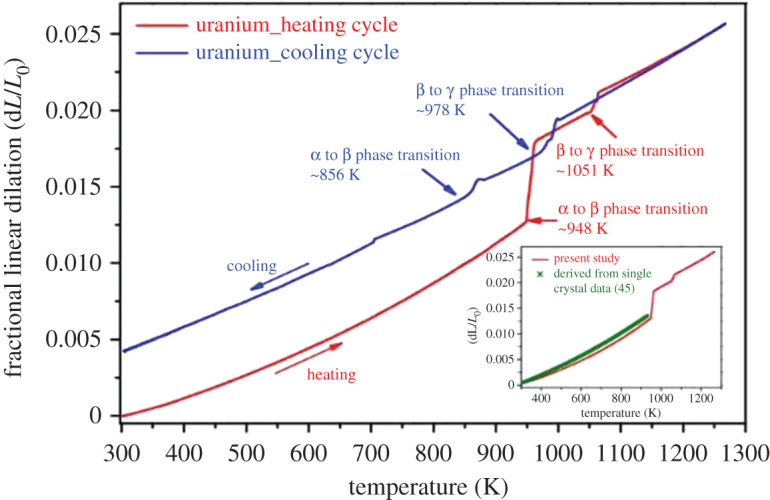
Linear thermal expansion observations of polycrystalline uranium metal during heating and cooling cycles [28].

The physico-chemical properties of uranium metal over a wide temperature range (25–1000°C) were recently evaluated [28]. In the solid state, uranium metal (melting point: 1132°C) can be found in three different phases: an orthorhombic phase (α-U, below 675°C), a tetragonal phase (β-U, 675–778°C) and a cubic phase (γ-U, above 778°C). Each phase exhibits different specific heat, thermal conductivity and density. Linear thermal expansion behaviour of polycrystalline uranium metal with respect to temperature is illustrated in figure 1. Specific heat rises exponentially with temperature for the α-phase and is temperature independent for the β- and γ-phase. The thermal conductivity of the α-phase rises linearly as a function of the temperature, while the thermal conductivity of the β- and γ-phase is not affected by temperature. Density for all phases decreases linearly with the temperature, with sharp declines corresponding to the *α* → β and β → γ transitions. The γ-phase presents high thermal conductivity, suggesting potential applications of cubic-phase uranium alloys for metallic nuclear fuel. A summary of these properties is presented in table 1 [28].

**Table 1.** Physico-chemical properties of uranium metallic phases [28].

phase	thermal stability range (°C)	lattice (space group)	specific heat (J g^−1^⋅K)	thermal conductivity (W m^−1^⋅K)	density^a^ (g cm^−^^3^)	IAEA recommended density (g cm^−^^3^)
α-U	<675	orthorhombic (cm)	∼0.12 (27°C) to 0.20 (675°C)	∼24 (27°C)–39 (675°C)	18.86 (27°C), ∼18.2 (675°C)	19.04 (27°C), ∼18.4 (675°C)
β-U	675–778	tetragonal (P4_2_/mnm, P4_2_nm or P4n2) [9]	∼0.18	∼40	∼17.8	∼18.1
γ-U	>778	cubic (unknown)	∼0.16	∼44	∼17.7 (778°C), ∼17.5 (1000°C)	∼17.9 (778°C), ∼17.8 (1000°C)

^a^Densities in this study were lower than the IAEA recommended values attributed to relatively higher carbon content, history of metallurgical processes and polycrystallinity.

When exposed to air, uranium metal oxidizes and forms a dark oxide layer. Finely powdered uranium metal is pyrophoric, and atmospheric control is required to manipulate it. Caution needs to be taken while manipulating uranium as it is toxic and can cause poisoning upon inhalation or ingestion [29]. It is noted that the toxicity of uranium is mostly chemical rather than radiological. Uranium metal is insoluble in alkalis and dissolves in acids. In concentrated HCl, U(IV) can be stabilized for long periods of time. The most common acid used for dissolving uranium metal is nitric acid [30,31]. Upon reaction with nitric acid, uranium metal oxidizes to U(VI), and depending on the nitric acid concentration, various uranyl nitrate species are found. For example in 4 M (HNO_3_), the main species in solution is UO_2_(NO_3_)_2_ [32]. Uranyl nitrates are commonly used as starting materials for the preparation of other uranyl compounds (e.g. UO_2_C_2_O_4_ and UO_2_Cl_2_) [33]. Tetravalent uranium, U(VI), and to some extent U(III) are the thermodynamically stable oxidation states of uranium in aqueous solution. Even if U(IV) and U(III) can exist in aqueous media, U(VI) is by far the most stable oxidation state.

## 3. Solid-state reactions

### 3.1. Magnesiothermic reduction

The preparation of uranium metal using magnesium metal was initially reported in 1893 by H. Moissan. Moissan prepared uranium metal from the reaction of Na_2_UCl_6_ with powdered magnesium metal at 400°C in a metallic tube [23]. The MTR of UF_4_ in a graphite crucible under argon atmosphere was first performed in 1942 [21]. This method mimicked the preparation of beryllium metal. While yield and quality of the metal were initially low, the process was continuously optimized owing to the demand of uranium metal. By early 1943, the reduction process, yields and quality of the metal had greatly improved such that it became the primary method for uranium metal production [21]. From 1893 to the present day, MTR has been a subject of intense research [8,23,34–40].

MTR is performed in a closed reaction vessel (reactor). In this method, UF_4_ is reduced by Mg metal (the mixture is referred to as the ‘charge’) at high temperatures in an oxygen-free atmosphere (equation (3.1)):

3.1 $${\mathrm{UF}}_{4}+2\mathrm{Mg}\to U+2{\mathrm{MgF}}_{2}.$$

**Figure 2.**
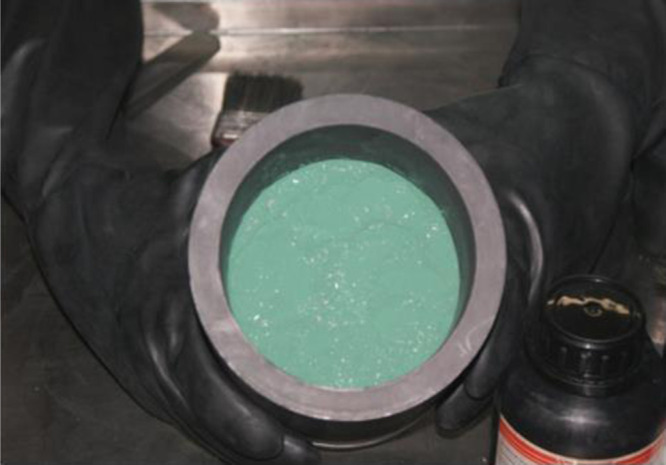
Experimental set-up for the preparation of uranium metal by MTR. Ten layers of UF_4_ and Mg metal (1815 ± 5 g total, 15% excess Mg) tapped in graphite crucible prior to reaction [8].

The reactor is occasionally lined with MgF_2_ to prevent contamination owing to reactor corrosion. The blended UF_4_ and excess (typically 10–15%) Mg metal charge are packed in the reactor, and the lid is protected with MgF_2_ powder and bolted to the reactor. The reactor is preheated in a furnace (570–620°C) [8]. After sufficient preheating, the temperature is raised, and the reaction is initiated. This firing with a sudden rise in temperature from the exothermic reaction [35,41] is observed using a temperature recorder. Owing to its high density, uranium metal settles at the bottom of the crucible allowing its separation from the slag and other reaction products, primarily MgF_2_; the quality of this separation is an important factor for yield [40]. A typical UF_4_/Mg load in a graphite crucible is shown in figure 2 [8]. Yields of approximately 80–96% have been reported depending on uranium recovery methods [8,40]. The most significant parameters on controlling firing time include the tap density of UF_4_, the moisture, the free acid content in UF_4_, the Mg particle size and the oxide presence in Mg [35].

The presence of oxygen and water in the system interferes with the reaction, creates impurities and decreases the yield of the reaction. For example, uranyl difluoride (UO_2_F_2_) and oxides are produced from the oxidation of UF_4_ with air [42–44]. For this reason, UF_4_ should be anhydrous and production routes that facilitate the formation of hydrated species should be avoided [44,45]. The presence of oxygen in the Mg metal will also cause the formation of uranium oxides [46]. Thus, it is important that the reaction is performed in the absence of oxygen and moisture [8].

During the reaction, small amounts of uranium metal could be trapped in the slag instead of settling to the bottom, lowering the yield. The dissolution of size-reduced slag discs in nitric acid and the combined melting of slag discs were used for metal recovery. Both methods require an appreciable mass of uranium for coalescence. The former poses complications owing to NO_x_ production and fluoride contamination, and the latter requires an additional cost-intensive set-up. The co-melting of slag discs was investigated as a third method and did not require additional set-up [40].

The co-melting of slag discs with the charge (i.e. UF_4_/Mg) uses the heat generated from the exothermic reaction. Thirty batches were co-melted using one slag disc each [40]. The slag discs (4–16 kg) were able to co-melt with the charges (approx. 200 kg ingots), and the best separations were observed in thin or light slag discs. The average recovery yield from slag discs was 92%, and the purity of the recovered metal was unaffected (as high as 99.95%) [47]. This method was shown to be simple and cost-effective and presents high potential for the uranium recovery from old stock. However, impurities from slag discs can build up in the metal product, and the amount of slag per batch is limited by thermodynamic constraints [40].

### 3.2. Calciothermic reduction

The preparation of U metal by calciothermic reduction was first reported in 1926. A corundum crucible was charged with UCl_4_ and calcium metal, placed in a steel bomb, and the bomb was evacuated and heated to the ignition temperature [21]. About three pounds of uranium metal were recovered upon cooling. The recovered metal was poorly characterized, though it appeared to be of good quality. The general equation for the reduction of UX_4_ (X = F, Cl) with calcium is shown in equation (3.2). The reduction of UBr_4_ with calcium yields CaBr_2_ (gaseous above 809°C) could become a preferred method if an economical preparation of UBr_4_ develops [48]. The preparation of U metal by calciothermic reduction has been studied by several authors [48–53]:

3.2 $${\mathrm{UX}}_{4}+2\mathrm{Ca}\to U+2{\mathrm{CaX}}_{2}.$$

The temperature achieved during the reduction of UF_4_ with calcium metal was calculated (2100°C), experimentally measured (2000°C) and was well above the melting point of CaF_2_ (1418°C). Because of the sufficient amount of liberated heat, preheating the charge was unnecessary, unlike the MTR process. These studies have shown the calciothermic reduction to be thermodynamically superior to the MTR [49]. However, since magnesium metal is cheaper than calcium metal and is more easily purified, the MTR method is primarily used in the industry [21,36,38].

**Figure 3.**
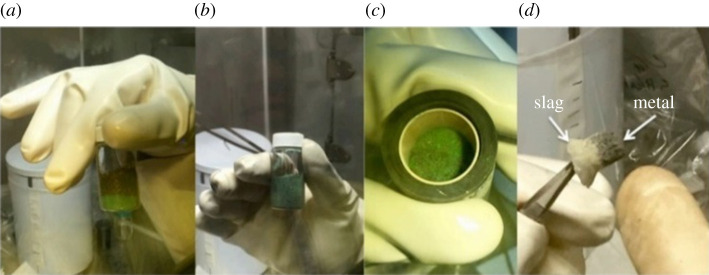
Preparation of uranium metal from calciothermic reduction. UF_4_, Ca and I_2_ sample: (*a*) prior to mixing, (*b*) after mixing, (*c*) in crucible, and (*d*) separation of slag and metal [53].

Calciothermic reduction has also been used for the separation of thorium from uranium. This experiment (figure 3) was performed to further investigate the reaction mechanisms (how thorium separates from uranium) and product properties (morphology features of metal and slag) as well as to better understand how Th associates with other elements (Fe, Al, Si) [53].

In that study, the Th-doped UF_4_ was produced by dissolution of UO_2_ with HF_aq_ and 2000 ppm of ThF_4_. The charge consisted of a mixture of UF_4_, Ca, and I_2_ inside of a MgO crucible. The charge was reduced using an induction heater in an argon atmosphere, and the resulting metal and slag were probed using SEM/EDS (scanning electron microscopy/energy dispersive X-ray spectroscopy). The surface of the slag contained few traces of O, F, Mg, Al, I and Ta. Analysis of the slag analysis indicated Th to consistently associate with Fe, Al and Si to produce sub-micron sized particles, resulting in the discovery of a new phase (Al_9-x_Fe_7-x_Th_2_Si_<1_). The Th-rich nanoparticles were extracted using a focused ion beam and analysed with SEM, transmission electron microscopy (TEM) and atom probe tomography.

### 3.3. Carbothermic reduction

The first study of the reduction of uranium oxide with carbon was reported in 1893. The reaction between sugar charcoal (40 g) and U_3_O_8_ (500 g) at high temperature produced a uranium ingot weighting 350 g (approx. 82.5% yield). The presence of carbon in the metal caused it to be brittle [21].

In the carbothermic reduction, carbon reacts with the uranium oxides to form CO gas and U metal (equation (3.3)). The formation of uranium carbide must be avoided, which is accomplished by lowering the metal activity (*vide infra*):

3.3 $$U_{x}O_{y}\;(s)+yC\;(s)\to xU\;(s)+y\mathrm{CO}\;(g).$$

**Figure 4.**
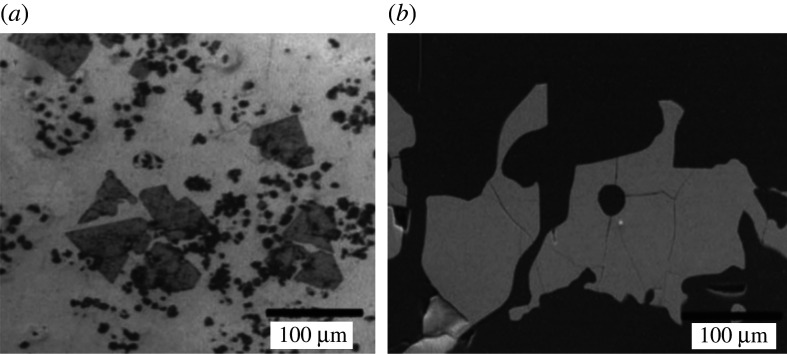
SEM images of (*a*) USn_3_ and (*b*) USi_3_ intermetallics.

In a more recent experiment, UO_2_ powder was mixed with graphite and amorphous carbon powder in either tin or silicon metallic baths [54,55]. The baths served to significantly lower the activity of the metal to accomplish the reaction. Graphite/alumina crucibles were filled with approximately 300 mg of the reaction mixture. Thermogravimetric analysis (TGA) and differential thermal analysis (DTA) were conducted up to 1670°C and revealed that silicon drove the reduction more effectively than tin. The reaction products were alloys and intermetallics (i.e. USn_3_ and USi_3_), and their morphologies were shard-like (figure 4). Quantitative EDS and XRD (X-ray diffraction) analysis indicated the presence of USn_3_, UC, UC_2_ and residual UO_2_ for the Sn process and high conversion (absence of oxides and carbides) for the Si process. Though yields were not reported, and isolation of the uranium metal was not discussed, the presence of uranium metal was supported by TGA/DTA, EDS and XRD analysis [54]. An advantage of this method is that the preparation of uranium halides precursors (i.e. UF_4_) is unnecessary as the reaction could be performed with oxides, though carbon contamination of the product is an inconvenience.

## 4. Electrochemical preparation

### 4.1. Electrochemical preparation in molten salts

The preparation of uranium metal in molten salt has been tied to the development of pyroprocessing of spent nuclear fuel (SNF) [56,57]. Pyroprocessing had been implemented in the US in the mid-1990s to treat sodium bonded SNF of the EBR-II (experimental breeder reactor) reactor. Fuel treatment used molten salts and liquid metals in an electrochemical operation. The molten salt was a LiCl-KCl eutectic, and two different electrorefiners (ER) were used: Mark-IV ER for driver fuel and Mark V-ER for blanket fuel [58–61]. Pyroprocessing of SNF in molten salts has several advantages, such as a reduced concern for criticality, flowsheet simplicity, minimization of liquid waste and the ability to handle shorter cooling times and high burn up of fuels [62].

The Mark-IV electrorefiner (batch size: 16 kg) used a 10 cm thick layer of molten cadmium and a 30 cm thick electrolyte layer, which was initially a LiCl-KCl eutectic with 5 wt% UCl_3_. After the treatment of SNF, the electrolyte became a mixture of fission product metal chlorides, transuranics and accumulated bonded sodium. The operating temperature of the electrorefiner was 500°C, and the anode and cathode assemblies rotated during the refinement process. The carbon steel anode basket was submerged into the electrolyte, and the fuel was electrochemically oxidized. The carbon steel cathode was also submerged where the reduced product was gathered [63]. Any uranium that falls or scrapes off the cathode was collected in the cadmium pool where it was dissolved and redeposited to the cathode. The Mark V electrorefiner was similar, but a cadmium pool was not used, and the uranium product was instead scraped off the cathode and collected in a basket [58].

Currently, electrowinning and oxide reductions are commonly used for SNF pyroprocessing. In electrowinning, actinide oxides are dissolved in alkali/alkaline earth halides, and the actinide metal is electrodeposited. In the reduction of actinide oxides in molten LiCl or CaCl_2_, the initial deposition of Li or Ca on the oxide is observed [62]. Both methods commonly use Pt, W, C or Ag electrodes, operate on a scale of several hundred grams of the molten salt [64,65] and are usually performed at 500°C or above (depending on electrolyte composition) [62]. Eutectic mixtures are used to keep operation temperatures as low as possible. For example, LiCl (m.p. = 605°C) and KCl (m.p. = 770°C) at a 3 : 2 molar mixture form a eutectic which has a melting point near 350°C [66]. A list of some molten salt eutectics used for uranium metal electrodeposition is presented in table 2.

**Table 2.** Salt composition, operation temperature and uranium species used for uranium metal electrodeposition.

salt composition (at. ratio)	species	operation temperature (°C)
3LiCl-2KCl	UF_4_	500 [64]
57.5LiCl-13.3KCl-29.2CsCl	UCl_3_	300–500 [66]
56.1LiBr-18.9KBr-25CsBr	UBr_3_	350 [65]
NaCl-KCl	UCl_3_	670–710 [67]
3LiCl-2KCl	UCl_3_	500 [68–71]
3LiCl-2KCl	UO_2_	450 [72]
NaCl-KCl or NaCl-KCl-LiCl	UCl_4_	800 or 700 [73]

Li_2_O/LiCl mixtures are generally used for the electrochemical reduction of actinide oxides (e.g. UO_2_). The cathode consists of a metallic basket loaded with the actinide oxide, and the anode is a platinum plate. Applying a reducing potential at the cathode (e.g. −1.52 V versus Ag/AgCl) will produce uranium metal. During the reaction, the oxygen ions (O^2−^) produced at the cathode diffuse through the molten salt, and O_2_ gas is released at the anode. Therefore, the reaction rate is largely affected by the diffusion of the O^2−^ ions in the salt.

Uranium tetrachloride was reduced in a 3LiCl-2KCl eutectic molten salt at 500°C for 17.5 h at a current of 0.1 A [64]. Uranium tetrachloride was added to the LiCl-KCl eutectic (U = 2.5–6.4 wt%), and cyclic voltammetry (CV) using a molybdenum metallic electrode was used to identify the oxidation states of the uranium species in the molten salt. Results indicated that the reduction of U(IV) occurred in two steps: a reversible U(IV) → U(III) reduction at –0.45 V versus Ag/AgCl and an irreversible U(III) → U(0) (metal) reduction at –1.52 V versus Ag/AgCl. Diffusion coefficients of U(IV) and U(III) were determined to be 0.91 × 10^−5^ cm^2^ s^−1^ and 1.39 × 10^−5^ cm^2^ s^−1^, respectively, at 500°C.

**Figure 5.**
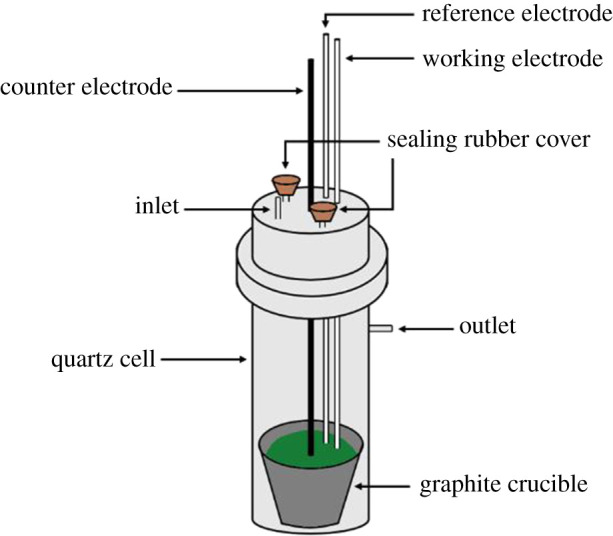
Electrochemical quartz cell with graphite crucible (adapted from [64]).

Galvanostatic electrolysis experiments were conducted using two types of cells: (i) stainless-steel cells with solid molybdenum cathodes and graphite anodes; and (ii) quartz cells (figure 5) equipped with liquid gallium cathodes and vitreous carbons anodes. Electrolysis in molten LiCl-KCl-UCl_4_ at 500°C for 17.5 h with a Mo working electrode and 1 h with a liquid Ga electrode was performed at a current of 0.1 A. XRD analysis indicated that uranium metal deposits (α-U) were produced on the inert solid plate, and a Ga_3_U intermetallic and U/Ga/UO_2_ impurities were found on the active liquid cathode [64].

### 4.2. Electrochemical preparation in room temperature ionic liquids

RTILs are salts that are liquid at/near room temperature (commonly defined as below 100°C) in ambient atmosphere [74,75]. Owing to their ionic conductivities, wide electrochemical potential windows, thermal stabilities, wide temperature ranges for the liquid phase and tunable solvent properties, RTILs are promising solvents for electrochemical applications [74] and have been proposed as solvents for nuclear fuel cycle separations [76]. Table 3 lists some RTILs used for uranium metal electrodeposition.

**Table 3.** RTIL composition, operation temperature and uranium species used for uranium metal electrodeposition.

RTIL	species	operation temperature (°C)
N-methyl-N-propylpiperidinium bis(trifluoromethylsulfonyl)imide (MPPiNTf_2_)	UO_2_	100 [62]
C_11_H_20_F_6_N_2_O_4_S_2_		
N-methyl-N-propylpiperidinium bis(trifluoromethylsulfonyl)imide (MPPiNTf_2_)	U_3_O_8_	100 [77]
C_11_H_20_F_6_N_2_O_4_S_2_		
N,N,N-trimethyl-1-butanaminium bis(trifluoromethylsulfonyl)imide ([Me_3_N^n^Bu][TFSI])	U(TFSI)_3_/UI_3_(THF)_4_	RT [78]
C_9_H_18_F_6_N_2_O_4_S_2_		

Electrochemical experiments in RTIL are conducted typically between room temperature and 100°C [62,74]. Ag/AgCl reference electrodes are commonly used owing to their stability and reproducibility at these temperatures [79]. RTILs enable electrodeposition of metals and alloys that were previously only accessible at high temperatures in molten salts [74]. Metals deposited in RTILs are of higher purity than those obtained in aqueous media because hydrogen evolution and hydride formation does not occur [74]. Because most RTILs are hygroscopic, water can accumulate and drying prior reaction is necessary. Physico-chemical properties of RTILs such as viscosity, conductivity, reactivity and solvating ability are largely affected by the presence of water. Humidity levels of the atmosphere must also be considered and controlled [75].

Electrochemistry studies of uranium in chloroaluminate-based RTIL were initially performed in the 1980s. U(IV) and U(III) were studied in acidic AlCl_3_-N-(n-butyl)pyridinium chloride melts (liquid below 27°C) [80]. Because of a limited electrochemical window and poor cathodic stability, the electrodeposition of uranium metal was not observed.

N-methyl-N-propylpiperidinium bis(trifluoromethylsulfonyl)imide (MPPiNTf_2_) is suitable for the electrodeposition of lanthanide and actinide metals as it offers a wide electrochemical window (approx. 5 V) and cathodic stability. MPPiNTf_2_ was used to investigate the electrodeposition of metallic uranium at room temperature [62]. A mixture of UO_2_ powder (0.48 g) and HNTf_2_ (2 g) was refluxed at approximately 100°C in water (30 ml). Uranium dioxide was shown to dissolve in HNTf_2_ (over 95% dissolved after 25 h). Following dissolution, the electrochemical behaviour of U(IV) in MPPiNTf_2_ at 100°C on platinum, glassy carbon and stainless-steel electrodes was investigated by CV. Cyclic voltammograms in MPPiNTf_2_ using platinum and glassy carbon electrodes were similar and exhibited four cathodic waves at approximately −0.7 V, −1.4 V, −2.2 V and −2.7 V versus Fc/Fc^+^. It was suggested that the first three waves correspond to the reduction of U(IV) to U(III). Meanwhile, cathodic waves were merged for the stainless-steel electrode. Controlled electrolysis at −2.4 and −2.8 V versus Fc/Fc^+^ on platinum and stainless-steel electrodes, respectively, resulted in metallic uranium deposition. The XRD patterns were consistent with the presence of α-U and with the absence of uranium oxide. The faradic yield on the platinum electrode was 80%. SEM/EDS analysis of the stainless-steel electrode reveals a uniform deposit of uranium metal as well as some cube-shaped crystallites [62].

**Figure 6.**
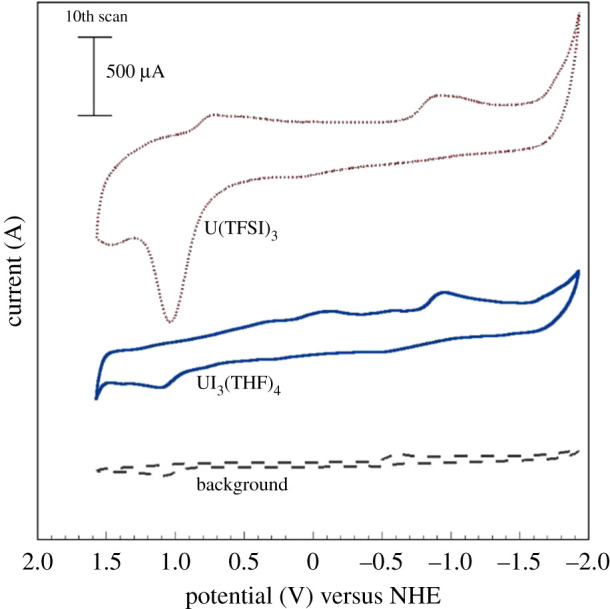
Cyclic voltammograms at 100 mV s^−1^ for U(TFSI)_3_ (top) and UI_3_(THF)_4_ (bottom) in [Me_3_N^n^Bu][TFSI] on gold electrode [78].

In trimethyl-n-butylammonium Bis(trifluoromethanesulfonyl)amide (e.g. [Me_3_N^n^Bu][TFSI]), U(TFSI)_3_ and UI_3_(THF)_4_ were electrochemically reduced in under argon atmosphere [81]. U(TFSI)_3_ was prepared from the reaction of KTFSI with UI_3_(THF)_4_ [81] in tetrahydrofuran (THF). Both U(TFSI)_3_ and UI_3_(THF)_4_ were dissolved in [Me_3_N^n^Bu][TFSI], and the respective solutions (5 ml) were analysed by CV using a gold electrode. The CV consisted of two reduction and two oxidation waves for both compounds. These waves correspond to U(IV) → U(III) and U(III) → U(0) reductions and U(0) → U(III) and U(III) → U(IV) oxidations. The deposition of uranium metal on a gold electrode was observed at −0.9 V (figure 6). It was proposed that the U(TFSI)_3_ species was reduced by a stepwise removal of n-bis(trifluoromethansulfonyl)imide (TFSI) ligands and that the free iodine released during the reduction of UI_3_(THF)_4_ could undergo further reactions with the uranium deposit and the gold surface.

**Figure 7.**
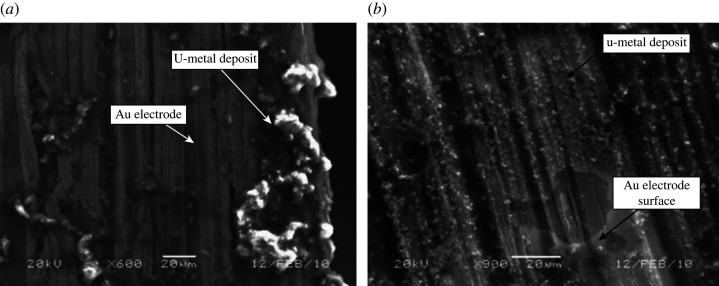
SEM images of the uranium deposit obtained after the electroreduction of U(TFSI)_3_ (*a*) and UI_3_(THF)_4_ (*b*) followed by annealing at 550°C for 5 h in a vacuum sealed glass tube [78].

EDS measurements of the deposits did not indicate any oxygen associated with the uranium, though some traces of low-Z elements associated with the TFSI ligands (i.e. C, H, N, S, O, F) were detected. Initial XRD measurements on the electrodes indicated the presence of α-U, though the diffraction pattern was weak owing to the poor crystalline nature of the deposit. Therefore, the samples were vacuum sealed in a glass tube and annealed for 5 h at 550°C. The glass tube was open in a glovebox under argon atmosphere and the XRD analysis on the reduction product of U(TFSI)_3_ indicated presence of α-U (wt.%: 2.03), for details on uranium phases, see §3. SEM/EDS measurements of both annealed samples were conducted, and trace contaminations observed previously were not detected (figure 7) [78].

### 4.3. Electrochemical preparation in acetic acid

Several studies have been reported in acetic acid for the preparation of uranium-mercury amalgams [47,82–85]. The experimental set-up usually consisted of an electrochemical cell equipped with a cation exchange membrane which maintains the pH of the catholyte at a constant value. A platinum anode and acidic solution were located in the anodic compartment and a mercury pool and a uranyl solution were contained in the cathodic compartment [86].

At the cathode, the uranyl ion (UO_2_^2+^) is electrochemically reduced to the metal (equation (4.1)) [82]. Following the reduction, uranium metal forms an amalgam with mercury, the uranium-mercury amalgam is treated at high temperature under vacuum, mercury is distilled away and uranium metal is recovered as a powder:

4.1 $${\mathrm{UO}}_{2}{\;}^{2+}+4H^{+}+6e^{-}\to U^{0}+2H_{2}O.$$

**Figure 8.**
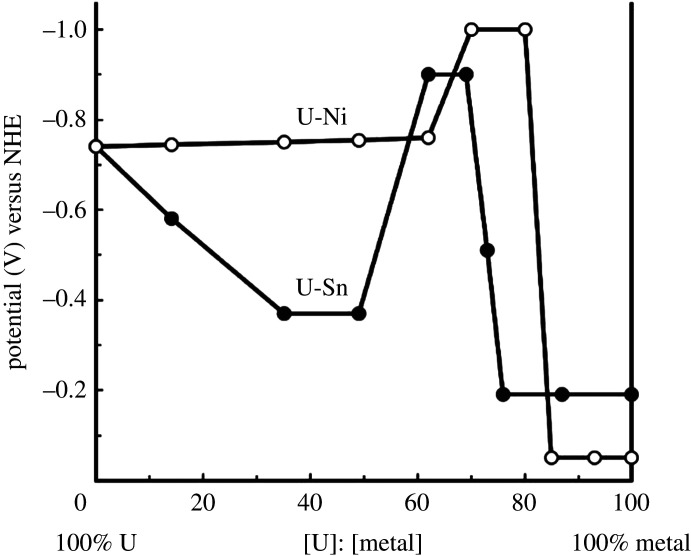
Dependence of redox potential of mixed amalgam on atomic ratios (adapted from [83]).

U-Ni and U-Sn alloys (i.e. UNi_5_ and USn_3_) were prepared in a two-compartment cell at room temperature in acetic acid from the electroreduction of uranyl acetate using a mercury cathode and platinum anode [83]. At −1.8 V versus NHE (standard hydrogen electrode), the catholyte was either nickel chloride or tin chloride solution. The amalgams were heterogeneous systems of intermetallic particles with yields up to 99%. The amalgams were processed into metals by thermal distillation of mercury in vacuum (up to 800°C) and intermetallic UNi_5_ and USn_3_ were identified by XRD and chemical analysis. A diagram of redox potentials of mixed amalgam as a function of U : Ni and U : Sn ratios is presented in figure 8 [83].

A uranium–mercury amalgam (1.9 mg U ml^−1^ Hg) was prepared in a two-compartment cell [84]. A platinum anode and an acidic sulfate solution were contained in the upper compartment, while a mercury pool cathode and a uranyl acetate solution (0.1 M, pH 2) were in the lower compartment. The potential was monitored with a calomel electrode. The cathode was maintained at −1.8 versus SCE (saturated calomel electrode) during electrolysis. The amalgam was converted to uranium metal by heating to 1300°C under vacuum. X-ray fluorescence spectrometry, metallography and thermal analysis were consistent with the presence of uranium metal [84].

A similar study at a kilogram scale was performed. A solid uranium–mercury amalgam with uranium content as high as 1.7 mg U ml^−1^ Hg was prepared electrochemically [82]. The experiment was repeated for current densities of 50, 100 and 130 mA cm^−2^. In each experiment, the anodic compartment was filled with Na_2_SO_4_ (1.5 l, 1 M) and the cathodic compartment with a uranium solution (25 mg U ml^−1^). The uranium solution was prepared by dissolving (NH_4_)_2_U_2_O_7_ (250 g) in HCl (130 ml, 12 M) and glacial acetic acid (320 ml), heating to 80°C and diluting to 8 l with water. A platinum plate/gauze and mercury pool (10 mm deep) were used as the anode and cathode, respectively, and a calomel reference electrode was used. The temperature was kept at 20–25°C and the potential at 10–11 V.

Electrolysis at higher current densities resulted in higher uranium recoveries in shorter times and higher current efficiencies, up to 88.2 wt% recovery in 3 h and 34.5% efficiency at 130 mA cm^−2^. In a 24 h operation at 100 mA cm^−2^, 1 kg of (NH_4_)_2_U_2_O_7_ was reduced to metal with an efficiency of approximately 24 kWh kg^−1^ U. Higher efficiencies could be observed at higher current densities (greater than 130 mA cm^−2^), but only if the concentration and temperature of the catholyte were kept constant. Gravimetric analysis was used to determine uranium content in the amalgam. The solid U-Hg amalgam was separated from its liquid portion, digested with 6 M HCl, precipitated with NH_3_, ignited and weighed as U_3_O_8_. Impurities detected (Ag, B, Cd, Cr, Cu, Fe, Mn, Mo, Ni, Th) were introduced by (NH_4_)_2_U_2_O_7_, and mercury content could be reduced to 10 ppm by heating to 1300°C under vacuum. These results suggested that 170 g of uranium could be reduced to the metal in aqueous media in 4 h with a 30% current efficiency and greater than 80% recovery [82].

The preparation of an U-Hg amalgam in acetic acid and sodium acetate was performed using U_3_O_8_ [47]. In the two-compartment set-up, the platinum anode was immersed in 1 M sulfuric acid. Though vigorous hydrogen evolution was observed at the mercury cathode, the pH was kept almost constant by H^+^ ions supplied through the membrane by the anode compartment.

In this study, U_3_O_8_ was dissolved in 6 M HCl containing a small amount of H_2_O_2_, evaporated to dryness, and the residue was dissolved in 0.5–1.0 M HCl. The U(VI) species in HCl was initially reduced to U(IV) at −0.6 V versus SCE, and amalgamation was performed at –2.0 to −2.3 V versus SCE. The amalgam was rinsed with water and ethanol, transferred to an alumina or magnesia crucible, treated at 1000°C, and heated above the melting point of uranium under vacuum. Thermal analysis confirmed the product to be α-U, and its density was measured to be 18.94 g cm^−3^ (theoretical value: 19.04 g cm^−3^; table 1). Yield as high as 99.4% was obtained, and the metal recovered had a purity greater than 99.95% [47].

This method was later applied for the preparation of neptunium metal, and the preparation of other actinide metals using a similar method was proposed [85].

### 4.4. Electrochemical preparation in organic solvents

In the aim to prepare uranium metal, Martinot *et al*. [87–89] investigated the electrochemistry of uranium in various organic media. The preparation of uranium metal by electrochemical reduction of U(III) was initially investigated in dimethylformamide (DMF) and in γ-butyrolactone/tetrahydrofuran (γ-BL/THF). In those experiments, UCl_3_, was used as the precursor [87].

Solutions of UCl_3_ (0.05 M in DMF and γ-BL/THF) were analysed by CV, though macroscopic amounts of the metal were produced only in γ-BL/THF. A W working electrode and Pt foil pseudo reference electrode were used. The CV curves were not well-defined, but a potential plateau between −2.0 and approximately 4.5 V versus Pt was observed. SEM characterization revealed the presence of amorphous uranium metal deposits on the W electrode. A product probably consisting of UCl_3_ with organic materials was observed. It was reported that uranium metal regularly plates the cathode at a current density between 20 and 40 mA cm^−2^. The Faradaic yields were 39% ± 1% at 20 mA cm^−2^ and 26% ± 1% at 30 and 40 mA cm^−2^, respectively.

In order to further investigate the use of organic solvents to prepare uranium metal, the electrochemical properties of U(IV) and U(VI) species (Cs_2_UCl_6_ and Cs_2_UO_2_Cl_4_) were studied in organic media containing tetrabutylammonium tetrafluoroborate ((TBA)(BF_4_)) [88]. U(IV) was studied in phenanthrene/(TBA)(BF_4_) (85/15 wt%), naphtalene/(TBA)(BF_4_) (90/10 wt%) and diphenyl/(TBA)(BF_4_) (86/14 wt%). U(VI) was studied in phenanthrene/(TBA)(BF_4_) (75/25 wt%), biphenyl/(TBA)(BF_4_) (83/17 wt%), naphtalene/(TBA)(BF_4_) (90/10 wt%) and phenanthrene/benzene (15/85 wt%) with approximately 0.4 M (TBA)(BF_4_). A Pt working electrode, a Pt foil reference and counter electrodes were used with a Pyrex cylinder electrochemical cell. The solutions containing U(IV) and U(VI) species were analysed in a glovebox by CV, chronoamperometry, sampled polarography and differential pulse polarography techniques.

Among the systems studied, the reduction of U(IV) and U(VI) in phenanthrene/(TBA)(BF_4_) demonstrated the most promising results for the preparation of uranium metal. The redox behaviour of Cs_2_UCl_6_ was studied by CV in phenanthrene/(TBA)(BF_4_) (85/15 wt%) at 142°C at a rate of 500 mV s^−1^. Although CV results indicate the formation of uranium metal, the metal could not be prepared by long-term potentiostatic electrolysis owing to the passivation of the cathode. When conducted with a mercury pool cathode, 12% of uranium present in the solution was recovered, with a Faradaic yield of 41%. The reduction of U(III) to uranium metal at the mercury pool cathode proved to be feasible.

The authors suggested that the phenanthrene/(TBA)(BF_4_) system could be used for the preparation of other actinide metals (Np, Pu, Am) using Cs_2_NpCl_6_, Cs_2_PuCl_6_, PuCl_3_ and AmCl_3_ as precursors [88].

Martinot *et al*. also investigated the electrochemical reduction of U(IV) in hexamethylphosphoramide (HMPA), dimethylsulphoxide, ethylene glycol dimethylether and propylene carbonate at 25°C, and at 127°C in HMPA dimethylsulphone (DMSO_2_) media. Using Cs_2_UCl_6_ as the precursor, macroscopic amounts of uranium metal were only produced in molten DMSO_2_ at 127°C. Uranium metal was prepared by the electrochemical reduction of a Cs_2_UCl_6_ (0.03 M) solution containing LiCl (0.1 M) at 127°C. At the end of the experiment, the platinum cathode was plated with ‘a powdery crust of the solvent decomposition products containing small metallic uranium dendrites and nodules together with reaction products originating from the conducting salt’. The presence of the metal was confirmed by XRD and the faradic yield for the uranium deposition was 17.8%. The deposition of metallic uranium in DMSO_2_ occurred at about −2.8 V versus Fc/Fc^+^. The authors mentioned that this technique could potentially be used for the electrochemical separation of La and U, as the half-wave potential of La (in Hg) was reported to be −1.7 V versus Fc/Fc^+^ [89].

## 5. Chemical preparation

### 5.1. Chemical preparation in acetic acid

Several studies focused on the chemical preparation of uranium metal in acetic acid using sodium as the reducing agent [90,91]. It was shown that U(VI) could be reduced in acetic acid using a sodium-mercury amalgam [90]. In this experiment, uranyl solutions were reacted with sodium amalgam to yield a uranium–mercury amalgam, which was then concentrated and thermally decomposed to uranium metal. The uranyl solution was prepared by the dissolution of UO_2_ in 6 M HCl containing small amounts of H_2_O_2_, followed by evaporation and redissolution in acetic acid. The total amount of the uranyl solution prepared was not mentioned. A sodium–mercury amalgam, prepared by electrolysis of NaOH with a Hg cathode, was added to the uranyl solution by both drop-wise and batch methods.

In the drop-wise method, the uranyl solution (0.0067 M, 150 ml) was placed in a reaction tower where the sodium amalgam (13 wt.%, 100 ml) was dripped from the top (100 ml/10–15 min) through a capillary. This resulted in a uranium amalgam of 0.2 g U/100 ml Hg. This process was repeated multiple times, each time with new sodium amalgam, after the reaction the unreacted sodium was dissolved from the resulting amalgam. The amalgam was concentrated in a glass flask by vacuum distillation of mercury at 350°C, where a composition of 15 g U/50 ml Hg was achieved. A total of 11 l of the uranyl solution and 7.5 l of the sodium amalgam were used.

In the batch methods, the uranyl solution (0.13 M, 25 ml) was shaken with sodium amalgam (0.85 wt.%, 10 ml) in a 250 ml separatory funnel. This resulted in a uranium amalgam composition of 0.5 g U/10 ml Hg. The reaction was repeated until a concentration of 19 g U/460 ml Hg was achieved after which the amalgam was concentrated to 19 g U/50 ml Hg using reduced pressure filtration where most of the uranium was retained on a glass filter. The decomposition of the resulting amalgams was conducted in a magnesia crucible inside a quartz cylinder attached to a vacuum system. Prior to loading the sample, the crucible was heated to 1200°C at 5 × 10^−6^ mm Hg and then cooled under argon atmosphere. The amalgam was heated under vacuum to 350°C to remove most of the Hg and then to 1500°C to recover uranium metal.

Uranium metal yields varied from 50% (batch method) to 80% (drop-wise method) and could be improved by recycling uranium in the aqueous phase. Two metal samples were prepared: the first sample (metal-1) was resistance heated at 1250°C and indicated poor agglomeration of the metal, while the second sample (metal-2) was heated at 1500°C and produced a satisfactory ingot. Both metals were electropolished. The XRD patterns of the metallic products were weak or broad but consistent with the presence of α-U. In both samples, mercury and sodium impurity (less than 5 and less than 15 ppm, respectively) were detected. Among the other impurities (Ag, Al, B, Cd, Cr, Cu, Fe, Mg, Mn, Ni, Si, Sn, V), only boron exceeded the allowable impurity concentrations for nuclear fuel [92,93] and was probably owing to contamination from an external source. Uranium content in metal-1 exceeded 99.5% and was not mentioned for metal-2.

**Figure 9.**
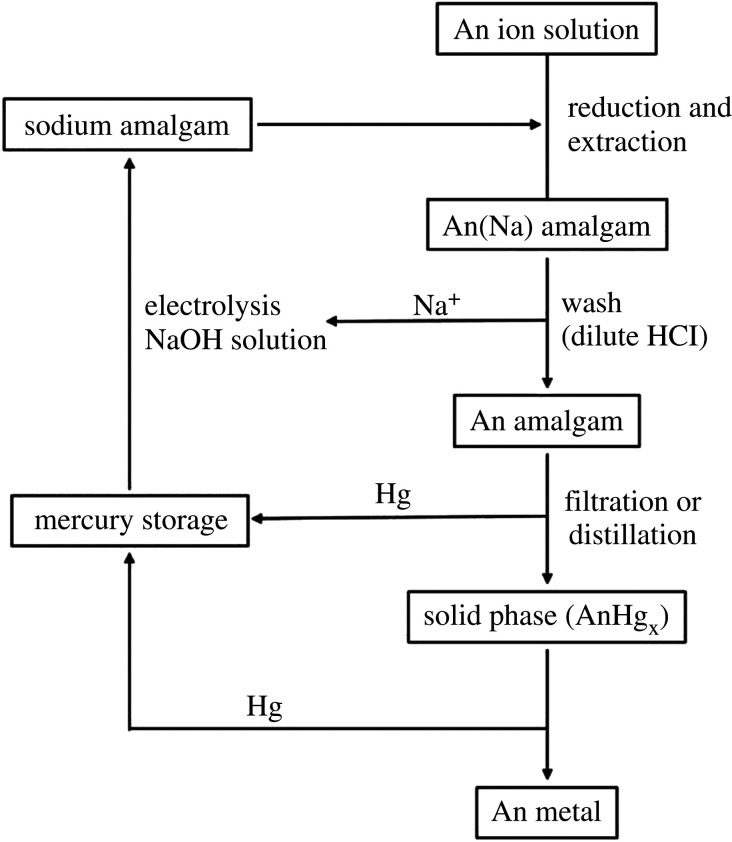
General reaction pathway for actinide metal preparation using chemical reduction in acetic acid (adapted from [90]).

A flowchart for the preparation of actinide metals using chemical reduction in acetic with Na is shown in figure 9. One advantage of this method is that the preparation of a uranium halide is unnecessary [90].

**Figure 10.**
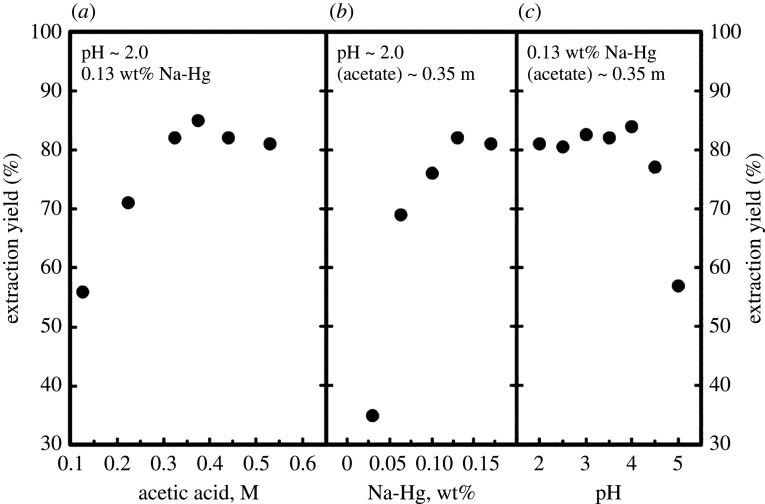
Representation of the uranium extraction yield as a function of (*a*) acetic acid concentration, (*b*) sodium amalgam concentration, and (*c*) initial pH (adapted from [91]).

A similar study was conducted with a uranyl acetate solution [91]. Uranyl nitrate was dissolved in acetic acid, evaporated to dryness and dissolved in distilled water. Sodium-Hg amalgam was added drop-wise to the uranyl acetate solution. Sodium was removed using 1 M HCl, and uranium was recovered by heating the amalgam in a water bath with 6 M HCl for several hours. The effect of pH and concentrations of acetic acid and amalgam on the extraction yield was investigated (figure 10). The optimal conditions were found to be a 0.35 M acetic acid, pH less than 4 and 0.13 wt% sodium in the amalgam. Extraction yields up to 87% were reported, though the recovery uranium metal was not performed [91].

### 5.2. Chemical preparation in tetrahydrofuran

**Figure 11.**
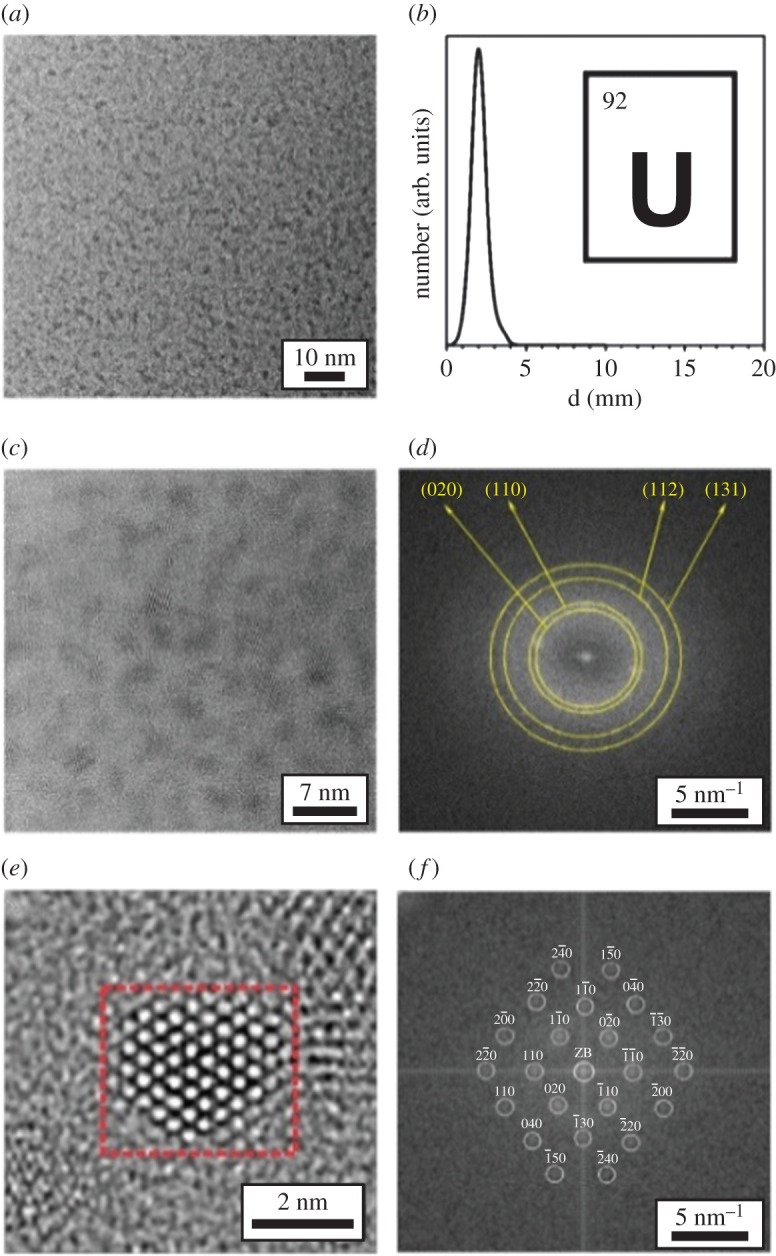
Transmission electron microscopy images of uranium nanoparticles. (*a*) TEM overview, (*b*) size distribution, (*c*) HRTEM (high-resolution transmission electron microscopy), (*d*) FFT analysis of particle ensemble, (*e*) HRTEM of single particle and (*f*) FFT analysis of red marked area [94] (https://pubs.acs.org/doi/10.1021/acsomega.7b0144). Further permissions related to this figure should be directed to the American Chemical Society.

Uranium metal nanoparticles were prepared by the reduction of UF_4_ with lithium naphtalenide (LiNaph, prepared *in situ*) in THF [94]. Chemical synthesis, sample handling and analytical characterizations were performed under inert conditions in a glove box. A solution containing Li (28 mg) and naphthalene (600 mg) in THF (10 ml) was added to UF_4_ (380 mg) in THF (15 ml) while vigorously stirred. A deep black suspension was immediately observed and indicated formation of uranium metal nanoparticles. The particles were centrifuged and dried in vacuum. TEM analysis (figure 11) revealed a particle diameter of 2.0 ± 0.5 nm, and fast Fourier transformation (FFT) analysis shows excellent agreement with the diffraction pattern of α-U. Yields were not reported [94].

This study is the first report of uranium metal nanoparticles preparation by chemical reduction. This method, which had also been used to prepare gadolinium metal, may provide a path for the preparation of other *f*-element metallic nanoparticles. The controllable milligram scale of this reaction is a benefit not shared with previously discussed methods, though there are limitations and requirements. Because uranium metal nanoparticles are highly pyrophoric, inert atmosphere conditions and proper safety precautions are required for their manipulation. Although the nanoparticles are stable in THF, they are highly reactive in other solvents (halogens, acids), and their interaction with alcohols (i.e. hexanol, octanol) could lead to hydrogen evolution.

## 6. Thermal decomposition

### 6.1. Arc melting

**Figure 12.**
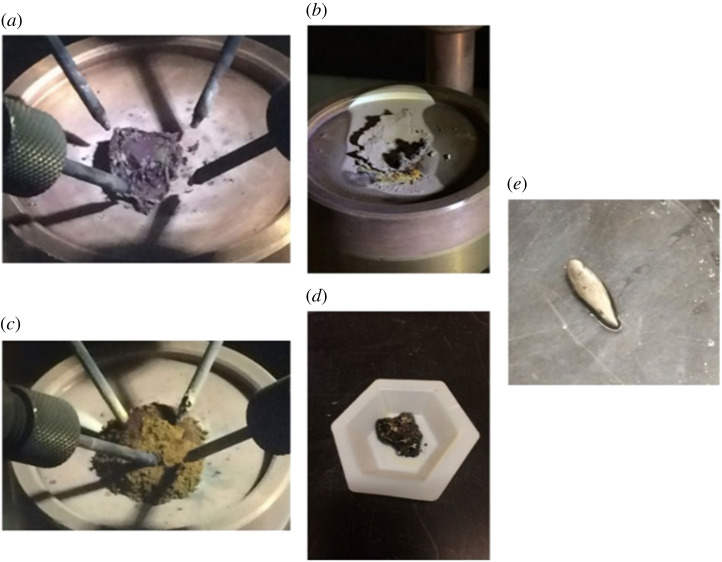
Preparation of uranium metal from the thermal decomposition of UI_3_ in an arc melter. (*a*) UI_3_ sample (2.11 g) on hearth, (*b*) after arc melting, (*c*) transformed to powder after extended time (greater than 1 h) under ultra-high purity argon, (*d*) after aged in air for several minutes, and (*e*) the recovered uranium metal (133.2 mg) [96].

A previous study has shown that arc melting could be used to decompose uranium samples. A previous study showed that the arc melting of UO_2_ produced a material with a UO_2–x_ (0.15 > x > greater than 0) stoichiometry; it was shown this material to be mixture of UO_2_ (main phase) and β-U metal (minor phase, 2–6 at.%) [95]. Recently, the thermal decomposition of UI_3_ (2.11 g) in a tetra-arc crystal furnace under argon atmosphere has been used to prepare metallic uranium [96]. The decomposition theoretically occurs at approximately 2100°C, a temperature higher than the sublimation point of UI_3_ (700–750°C) and the melting point of uranium metal (1132°C). The colour of the sample changed from jet-black to purplish-black upon evacuation of the arc chamber. Molten metallic iron was used as the iodine getter, and the arc was maintained at 20 to 30 A during the decomposition. The arc interacted with the sample and the reaction was initiated. Figure 12 shows the evolution of the UI_3_ sample during the experiment. Unreacted UI_3_ that became liquid upon ageing was rinsed to recover uranium metal (133.2 mg, approximately 20% yield). XRD shows the recovered product to be α-U, (*a* = 2.85286 Å, *b* = 5.86614 Å and *c* = 4.95692 Å), and density measurements indicated the density of the metal to be 19.2 g cm^−3^ (theoretical value: 19.04 g cm^−3^; table 1). Iodine impurities were determined to be less than 1 wt%. The lack of control and uncertainty of the achieved temperature was the primary challenge of this method. The authors have noted that the short contact time owing to a lack of conductivity between the arc and the sample caused only parts of the sample to reach sufficient temperatures [96].

### 6.2. Laser induced

**Figure 13.**
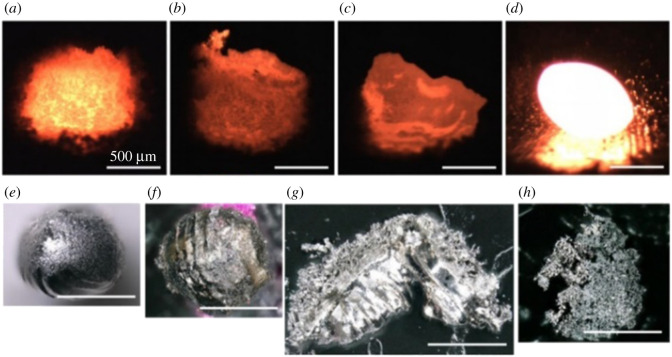
Preparation of uranium metal from the thermal decomposition of UN using a laser. *In situ* observations of UN laser heated on a W plate. (*a*) UN powder irradiated under high vacuum using a defocused beam for 30 s at 10 W laser power, (*b*) 30 s at 20 W laser power, (*c*) 30 s at 50 W laser power, and (*d*) using a focused beam at 50 W laser power. Reaction products after 900 s treatment in (*e*) high vacuum, (*f*) argon, (*g*) 95% argon/5% nitrogen, and (*h*) nitrogen environments (500 µm scale bars) [97].

Laser-induced thermal decomposition of UN (95% purity [65]) was performed using a 1070 nm, continuous wave fibre laser (YLR500-AC, IPG Photonics) [97]. Uranium mononitride thermally decomposes to uranium and nitrogen gas at 2500–2850°C [98]. Because nitrogen is formed and can react with metallic uranium, its presence in the atmosphere can limit the production of metal. A thick layer (300 µm) of UN was spread on a tungsten plate, and laser powder bed fusion experiments were performed under high vacuum, ultra-high purity argon, 95% argon/5% nitrogen and nitrogen atmospheres. The laser repeatedly scanned a 1 × 1 mm region of the UN powder at 1000 mm s^−1^. *In situ* observations of the experiment are presented in figure 13. Reaction yields of up to 92.2% were reported. The resulting metallic sample was characterized by optical microscopy, SEM and single crystal XRD. The sample produced under nitrogen was analysed by powder XRD. The XRD pattern was consistent with the presence of α-U and a purity of 96.2% was reported. The experimental results confirmed the dependence of nitrogen partial pressure on the amount of uranium metal produced as determined with computational methods [97].

In addition, the formation of U metal has been observed from laser-induced thermal decomposition of a 300 µm thick layer of UI_3_ spread over a stainless-steel substrate in an ultra-pure argon atmosphere [99]. This also resulted in the production of gaseous I_2_ which must be evacuated from the chamber. Given that the high-power laser irradiation induced melting of the stainless-steel substrate, critical care should be given in selecting compatible substrate materials. Attempting to minimize the laser pulse and temperature will only lead to vaporization of UI_3_ materials without any conversion to U metal, as UI_3_ does not decompose until 2100°C [96].

## 7. Radiochemical reduction

Radiochemical methods have been widely used for the preparation of alloy nanoparticles [100]. For example, U-Ln (Ln = La, Eu) [101] and M-N (M = Ag, Au, Ce, Cu, Ni, Pt) (N = Ni, Pd, Pt, Ag, Fe, Ru) metallic nanoparticles were prepared by gamma, UV, X-ray, laser and electron beam irradiation [100].

Gamma irradiation in aqueous media is the most common radiochemical method used for the preparation of metallic nanoparticles [102]. The gamma radiolysis of water produces several molecular and radical radiolytic products (i.e. $e_{\mathrm{aq}}^{-}$, H_2_, OH^•^, H^•^, H_3_O^+^) [103]. Among those radiolytic products, the hydrated electron ($e_{\mathrm{aq}}^{-}$, *E*^0^ = −2.58 V) [104] can reduce U ions to the metal. The radiolytic yield for the hydrated electron by gamma irradiation is approximately 2.7 × 10^−7^ mol J^−1^ [105].

Uranium metal nanoparticles were prepared from the gamma irradiation (^60^Co source) of U(IV) in aqueous media [101]. A solution of UCl_4_ (12.5 ml, 0.004 M) was prepared in deionized water with methanol, sodium citrate (0.1 M) and polyvinyl alcohol (0.1 M). Citrate ions and polyvinyl alcohol were used as stabilizers to affect particle size and shape. The vessel was purged with argon, and the solution was irradiated at a dose rate of 350 rad s^−1^ for 24 min and twice more for 6 min each at the same dose rate to ensure reduction completion. This was about five times the dose needed to reduce U(IV) to the metal. UV-visible spectroscopy and TEM analyses were performed after each irradiation and after 7 d.

**Figure 14.**
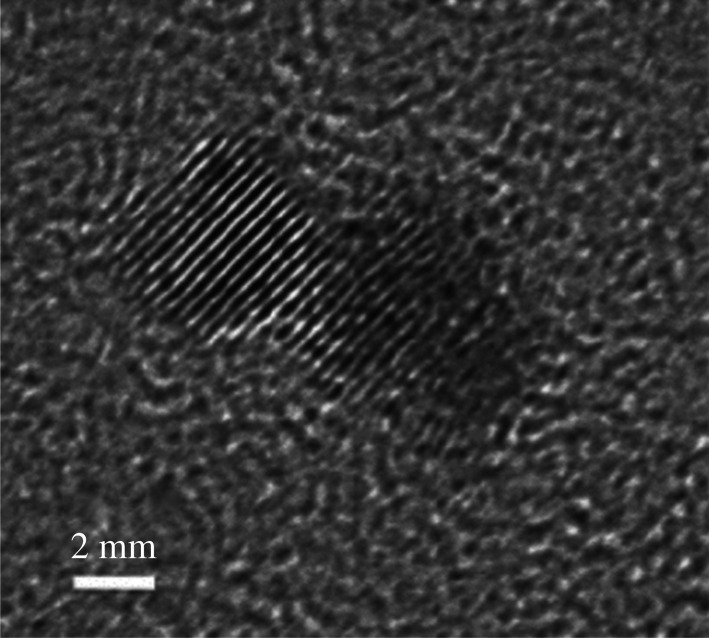
TEM image of uranium nanoparticles (6 nm average diameter) prepared from gamma irradiation of UCl_4_ [101].

UV-visible spectroscopy measurements of the solution after the first irradiation indicated a peak narrowing (250 nm) consistent with the formation of nanoparticles. The consistency between each spectroscopic measurement indicated the completion of the reduction to uniform particles. After 7 d, peak broadening was observed, which indicated agglomeration of nanoparticles to larger and varying particles and clusters. The appearance of a peak at 360 nm indicated the formation of UO_2_ nanoparticles. The average particle size (approx. 6 nm) was determined using TEM (figure 14). Selective area diffraction analysis indicated lattice spacing of 2.99 Å {0 2 0}, 2.59 Å {1 1 0}, 1.89 Å {0 0 2} and 1.61 Å {1 3 0} consistent with the presence of α-U. The yield of U metal was not reported, but the reduction was reported to go to completion according to the UV-visible spectroscopy measurement.

## 8. Discussion

Uranium metal can be prepared by diverse methods: solid-state high temperature reactions, electrochemical, chemical and radiochemical methods, and high temperature reaction using laser or arc melter. The key experimental parameters, yields and morphologies are summarized in table 4.

**Table 4.** Key experimental parameters across discussed methods. (N/A, not applicable; N/R, not reported; RT, room temperature.)

method	uranium precursors	reducing agent redox potential	scale	uranium metal yield (%)	temperature (°C)	morphology
magnesiothermic reduction in MgF_2_	UF_4_	Mg	g-kg	∼80, 92–96 from slag	1600–1700	solid
calciothermic reduction in CaF_2_	UF_4_, UCl_4_	Ca	10 g	N/R	1100	solid
carbothermic reduction	UO_2_	C	300 mg	N/R	1670	shard-like (intermetallic)
electroreduction in molten salt	UCl_3_	−1.52 V	g	N/R	500–600	acicular/dendritic
electroreduction in RTIL	UO_2_, UI_3_(TH)_3_ U(TFSI)_3_	−2.4–2.8 V	0.08 M (mg)	N/R	RT-100	amorphous
electroreduction in acetic acid	uranyl acetate (NH_4_)_2_U_2_O_7_ U_3_O_8_	−1.8–2.3 V	g	88–99	RT Mercury distillation:800	powder (from amalgam)
electrochemistry in organic solvents	UCl_3_, Cs_2_UCl_6_, Cs_2_UO_2_Cl_4_	−2.8 V	0.05 M (mg-g)	N/R	RT	amorphous
chemical reduction in acetic acid	UO_2_, UO­_2_(NO_3_)_2_	Na	10–1300 g	∼8	RT (amalgam decomposition: 1500)	powder (from amalgam)
chemical reduction in THF	UF_4_	LiNaph	380 mg	N/R	RT	nanoparticles
thermal decomposition by arc melting	UI_3­_	N/A	2.11 g	20%	2100+	solid
laser-induced decomposition	UN		∼1.5 g	96.2%	2500–2850	monolith
gamma irradiation in aqueous/alcoholic/citrate media	UCl­_4_	e_aq_^−^	4 mM	N/R	RT	nanoparticles

Solid-state high temperature reactions are used industrially to prepare U metal. These methods are reproducible, produce uranium metal in high yields approximately 80–96% [8,40], high purities (as high as 99.95%) [28], and can be scaled up to hundreds of kilograms [36]. These methods could be challenging to transpose at the laboratory scale owing to the highly exothermic behaviour of the reaction, the need for high temperatures (1100–1700°C) and apparatuses appropriate for thermite-type reactions and the difficulty to scale to milligram-gram amounts owing to the reliance on the thermal energy of the reaction to keep the slag molten for consolidation of uranium metal.

Uranium metal can be prepared by electrochemical methods in a variety of solvents including molten salts, RTIL, acetic acid and organic solvents.

Technical challenges include the use of high temperatures for preparations in molten salts, the manipulation of a toxic mercury amalgam for preparations in acetic acid, the availability of a specific uranium precursor for preparations in RTIL and the reactivity of the reduced U species in organic solvents. In chloride molten salts, the operating temperature (300–710°C) is dictated by the composition of the salt, while in RTIL, organic solvents and acetic acid preparation are performed at room temperature. Poor yields, lack of potential control, and the need to maintain stability in corrosive environments are issues associated with the use of molten salts.

Preparations in RTIL also require the use of a uranium species that could be challenging to prepare (i.e. U-TFSI). In RTIL, the preparation of U metal typically required long time periods (hours to days) primarily owing to high viscosity of the media that make diffusion of the uranium species at the electrode slow. Depending on the RTIL, metal deposits must be annealed for purification. Electrochemical reductions in acetic acid using mercury pool electrodes present alternatives to molten salts or RTIL. The reactions could be performed at room temperature, and the preparation of a halide precursor is unnecessary. Yields and purities of the metal as well as the ability to scale down volumes are unclear, and the need to distill a uranium–mercury amalgam at high temperature is the main drawback of this method. The preparation of U metal in organic solvents is successful only in a small number of systems (i.e. γ-BL/THF, phenanthrene/(TBA)(BF_4_), DMSO_2_). For most of the systems studied, it was observed that uranium reduced species interacted with the solvents thus limiting the formation of U metal. Further electrochemical studies in organic media are needed to optimize these methods. Owing to relatively slow rates limited by the electrode surface area and the viscosity of the solvents, the timescales of these reactions is typically from hours to days. For most of the electrochemical preparations (except in acetic acid), the reaction yield was not reported. Owing to these constraints, the preparation of large amounts of uranium metal by electrochemical methods is challenging and time consuming [106–108].

Uranium metal was prepared by chemical reduction in acetic acid and in THF using alkali metal (Na, Li) as the reducing agents. Chemical reduction in acetic acid exhibits the same inconvenience as the electrochemical method: the production of a uranium–mercury amalgam that needs to be distilled. This method also requires a specific set-up for the formation of uranium amalgam and high temperature for the distillation of mercury. In these reactions, the yield varied from 50% to 80% depending on the method used (drop-wise versus batch), and repeated runs were necessary to achieve high yield. These reactions bypass the need for a uranium halide and the procedures were transposed to other actinides [90,91]. Chemical reductions in THF using a Li complex were conducted at the milligram scale and produce U metal nanoparticles with high purity that can be very challenging to handle outside of a glovebox. Uranium metal nanoparticles could also be prepared via gamma irradiation of U(IV) in water/alcohol; yields for this method were not reported, and experimental set-ups required gamma irradiators that are available only in few radiological facilities.

Thermal decomposition using arc-furnace and laser have been the most recent methods developed for the preparation of uranium metal. Those methods can be deployed in a well-controlled environment in the laboratory on the mg scale. The preparation of uranium metal using arc melting has significant advantages: it can be accomplished on a short timescale (seconds to minutes), and the metal produced exhibits a high purity [96]. The laser-induced reaction occurs at temperatures hundreds of degrees higher than the arc-furnace method (greater than 2500°C versus approximately 2100°C), and the thickness of the high purity uranium layer is limited by vapour depression depth induced by the laser. Using uranium nitride as a precursor, the byproduct of the reaction (i.e. nitrogen gas) is relatively simple to handle. Furthermore, the ability to predict optimal experimental conditions using thermodynamic calculations has been demonstrated [97]. The ability to predict and achieve optimal experimental conditions as well as the short reaction times (i.e. minutes) is a benefit not shared with other synthetic techniques. However, special equipment (i.e. arc melter and laser), high temperatures and inert atmospheres are essential for these reactions, and the lack of temperature control may hinder yield and purity of the metal.

Based on the current state of the art, the authors would like to highlight two promising methods that could be simply deployed in a laboratory but needs further development: (i) the electrochemical reduction of uranium in RTIL and (ii) the chemical reduction of U species in THF using Li. Future work in RTIL should focus on the identification of suitable U precursors that are simple to prepare and exhibit large solubility and stability in the RTIL and on the understanding of their electrochemical behaviour.

Future work related to the chemical reduction in THF should focus on the development of methods to prepare and manipulate U nanoparticles without the use of a glovebox. These methods could use standard Schlenk techniques for their preparation and pressing into pellets in glove bags for their manipulation. It is noted that the study of uranium nanoparticles could lead to interesting physico-chemical properties [109].

Finally, following their optimization using uranium, those two methods could be transposed to transuranics for the preparation of materials that are challenging to obtain from commercial sources (i.e. Np metal).

## Data Availability

All data presented in here are available in the literature and are properly cited throughout the text.
